# Group cognitive behavioural therapy with compassion training for depression in a Japanese community: a single-group feasibility study

**DOI:** 10.1186/s13104-017-3003-0

**Published:** 2017-12-04

**Authors:** Kenichi Asano, Haruna Koike, Yuriko Shinohara, Hiromi Kamimori, Akiko Nakagawa, Masaomi Iyo, Eiji Shimizu

**Affiliations:** 1 Research Center for Child Mental Development, Chiba University, 1-8-1 Inohana, Chuo-ku, Chiba, Japan; 2 Cognitive Behavioral Physiology, Graduate School of Medicine, Chiba University, 1-8-1 Inohana, Chuo-ku, Chiba, Japan; 3 Department of Psychiatry, Graduate School of Medicine, Chiba University, 1-8-1, Inohana, Chuo-ku, Chiba, Japan

**Keywords:** Compassion mind training, Compassion focused therapy, Group cognitive behavioural therapy, Depression, Community, Feasibility, Acceptability, Intervention

## Abstract

**Objective:**

Depression is a representative mental problem, and more than 350 million people are suffering in the world. Cognitive behavioural therapy (CBT) in individual or group formats is mainly recommended in major guidelines. However, patients with high self-criticism have a poor response to CBT. To treat such patients, psychotherapies focusing on compassion are gaining attention. Although trials have begun to be reported, there are relatively few studies examining the effectiveness of group CBT with compassion work for managing depression. The purpose of this study was to evaluate the feasibility and acceptability and the estimate effect size of group CBT with compassion training for future controlled studies.

**Results:**

Fourteen participants were enrolled in the trial, of which 13 completed the intervention, and 12 completed a 6-month follow-up assessment. Participants received a 1 h group-based CBT with compassion training session every week for 10 weeks. The effect of the intervention on participants’ Beck Depression Inventory score was examined using a general linear mixed model. This analysis showed an effect size of d = 1.12 at post intervention and d = 0.92 at 6-month follow-up. Group cognitive behavioural therapy with compassion training for depression shows feasibility and acceptability in a Japanese community.

*Trial Registration* UMIN000015007

**Electronic supplementary material:**

The online version of this article (10.1186/s13104-017-3003-0) contains supplementary material, which is available to authorized users.

## Introduction

Depression is a representative mental problem, and more than 350 million people are suffering in the world [1]. Cognitive behavioural therapy (CBT) is the primary recommended treatment for depression in major guidelines [2, 3]. A meta-analysis indicated that group CBT is useful for patients with depression [4]. However, patients with personality difficulties or high self-criticism have a poor response to cognitive therapy [5, 6].

To treat such patients, compassion focused therapy (CFT) has been developed [7]. In relation to CBT, compassion mind training (CMT), that is group intervention to develop a compassionate mind, can be effective when combined with CBT [8]. However, studies examining the effectiveness of an established combined manual for group CBT (GCBT) with compassion work are not enough.

This study aimed to (a) evaluate the feasibility and acceptability of group-based CBT with compassion training, and (b) estimate its effect size on depression (measured by the Beck Depression Inventory) for future controlled studies.

By combining compassion training with CBT, we included the components of treating perfectionism, shame, and self-criticism. A meta-analysis revealed that all perfectionism dimensions had positive relationships with follow-up depressive symptoms after controlling for baseline depression and neuroticism [9]. Some reports have revealed that shame is related to depression or predicts depressive symptom [10, 11] via traumatic memories that prevent people from regulating emotions [12, 13]. Self-criticism can also predict the reoccurrence of major depression and relates to the severity of depressive symptoms in patients with major depressive disorder [14, 15]. According to the CFT perspective, self-criticism seems to be safety behaviour to avoid key fears like shame, powerlessness, or rejection. Reducing self-criticism can avoid unwanted results like worthlessness or losing the sense of self, thereby stopping a vicious cycle as illustrated in Additional file 1 [16]. These three tendencies contribute to developing and retaining depression, and intervention by using compassion can be expected to be a more effective treatment.

## Main text

### Method

#### Study design

**Fig. 1:**
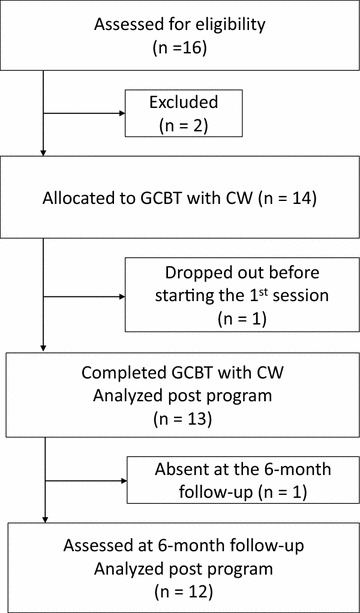
Flow of the participants in this study

This study adopted a single-group design (Fig. 1) and was conducted at the mental and emotional health center in Chiba City, Japan. All participants provided a written informed consent. All procedures were performed in accordance with the Helsinki Declaration. Required ethical approval was obtained from the Ethics Committee of the Chiba University Graduate School of Medicine (#1872). This study was conducted according to the Transparent Reporting of Evaluations with Nonrandomized Designs (TREND) statement [17]. The participants were enrolled from December 2014 to March 2016.

#### Participants

Participants were recruited using leaflets placed at medical institutions in Chiba City and web-based advertisements. Since all participants continued treatment from their psychiatrist, they were required to obtain permission from their psychiatrist prior to study enrolment.

The eligibility criteria were a primary diagnosis of major depressive disorder according to the diagnostic and Statistical Manual of Mental Disorders IV (DSM-IV) for individuals aged 20–65 years. To ensure that the study population reflected the routine clinical practice, comorbid diagnoses were permitted only if the problem was secondary. The exclusion criteria were as follows: psychosis, personality disorders, bipolar disorder, high risk of suicide, substance abuse or dependence in the past 6 months, unstable medical condition, pregnancy, or lactation.

All patients were asked to bring a referral form from their psychiatrist along with a confirmation of their treatment history. They were then interviewed and evaluated by a psychiatrist for the eligibility and exclusion criteria by the Mini-International Neuropsychiatric Interview (M.I.N.I.) [18]. The patient characteristics (gender, age, age of onset, duration of major depression, employment status, marital status, and educational background) were inquired in the referral form and interview for evaluation by the psychiatrist.

#### Interventions

Participants received 1-h of GCBT with compassion training every week for 10 weeks with a maximum of five patients per group. The therapist was a clinical psychologist, and the co-therapist was a nurse or psychologist with a master’s degree who received CBT Training course at Chiba University. To confirm therapists’ adherence to the protocol, peer supervision was held weekly using the checklist. The checklist was confirmed by the manual recordings written by the co-therapist after each session since no audio or video recordings were permitted by the city government. If the contents listed in the checklist were carried out, it was regarded as adhered. The therapist did not receive specific training for compassion works.

The GCBT with compassion training was based on a self-help book on CBT and compassion training compiled using free access resources [19, 20]. The program consisted of ten sessions as follows: introduction to GCBT and psycho-education of emotion (Session 1); instructions on self-monitoring as per the CBT model when distressed (Session 2); behavioural activation (Session 3); behavioural activation and monitoring of cognitions when distressed (Session 4); challenging one’s own negative cognitions (Session 5 and 6); psycho-education for perfectionism (Session 7); working with shame and self-criticism (Session 8); recalling memories of compassion (Session 9); compassion letters to self and other participants and relapse prevention (Session 10).

#### Outcomes

Outcomes were measured before and after the program, and during a 6-month follow-up.

The primary outcome measure was the Beck Depression Inventory-II (BDI-II), which is the most popular self-report questionnaire and has been used as an inclusion criterion in meta-analyses to assess depression severity [21, 22]. The BDI-II contains 21 items rated on a 4-point Likert scale ranging from 0 to 3. The score on the Japanese version of the BDI-II is classified as follows: 0–13 as minimal, 14–19 as mild, 20–28 as moderate, and 29–63 as severe. The Japanese version of the BDI-II has been standardised and demonstrates excellent reliability and validity [23].

The secondary outcome was the Self-Compassion scale in Japanese (SCS-J) [24]. The original version of this scale contains 23 items rated on a 6-point Likert scale ranging from 1 to 5 [25]. The SCS-J has been standardised and has demonstrated acceptable reliability and validity as a self-report questionnaire. The higher the total number of points, the higher the self-compassion.

#### Evaluation of feasibility and acceptability

Feasibility was evaluated by the rate of intervention completion, face-to-face interviews of participants, and adherence to the session checklist in peer supervision (see Additional file 2). The interviews were conducted within 2 weeks of completing the session by an independent officer of Chiba city. The participants were asked questions about satisfaction, intent to continue use, and improving methods in interview. If there was any dropout, the officer asked the reason via phone if it was possible.

#### Analyses

Descriptive statistics were calculated for outcomes. To evaluate the change of outcomes, intent-to-treat linear mixed model was used for both outcomes assuming that the missing values occurred randomly. They included time as the fixed effect and participant as a random effect. Further, restricted maximum likelihood method was used, and Cohen’s *d* was employed to calculate the effect size.

All statistical analyses were performed using a graphical user interface for R [26]. A significance level of *p* < 0.05 was adopted.

The answers to the interview questions about satisfaction and intent to continue the use were coded as ‘yes’ or ‘no’. Improving methods suggested by patients were categorised by the interviewer.

#### Sample size

Since this study was conducted as a community service, the sample size was not statistically determined. The maximum number of participants was 15 for whom the community was able to offer GCBT.

### Results

#### Characteristics of participants

Sixteen participants were screened, and fourteen of them were eligible for participation and enrolment. There was only one dropout before starting the first session. The demographic and clinical characteristics of the enrolled participants are shown in Table 1. Only one participant could not be contacted for the 6-month follow-up evaluation, and the sample size at the 6-month follow-up was 12.

**Table 1:** Characteristics of the participants

Demographic variables	Value
Gender	
Male, N (%)	10 (71)
Age (years), mean (SD)	40.5 (9.54)
Median	42
Range	28–56
Age of onset (years), mean (SD)	35.21 (9.32)
Median	39
Range	19–47
Duration of major depression (years), mean (SD)	5.28 (3.38)
Median	5
Range	2–15
Employment status, N (%)	
Employed full-time, temporary part time work	2 (14)
Employed full-time, suspended from work	2 (14)
Employed part-time	3 (21)
Unemployed	7 (50)
Marital status, N (%)	
Single	6 (43)
Married	8 (57)
Educational background, N (%)	
High school	5 (36)
≥ 3 years of college/university	9 (64)

#### Comorbidity

Based on the M.I.N.I., all participants were diagnosed with major depression; two participants had comorbid social anxiety disorder, and one participant had comorbid panic disorder.

#### Changes in outcomes

Table 2 shows the changes in outcome before and after the program, and at the 6-month follow-up assessment. The comparison was made using a regression model with mixed effect over time as fixed effect and patient as a random effect. The effect of time was significant on the BDI-II (*p* = 0.039) but not significant on the SCS-J (*p* = 0.51). The effect size of the BDI-II was 1.12 at post-intervention and 0.92 at the 6-month follow-up. The effect size of the SCS-J was − 0.46 after intervention and − 0.34 at 6-month follow-up. The BDI-II score decreased from 23.78 to 13.61 after intervention, which corresponds to a clinical improvement from moderate to minimal depression. At the post-intervention period, the response rate was 38% (5/13), and the remission rate was 46% (6/13). At the 6-month follow-up period, the response rate was 45% (5/11), and the remission rate was 45% (5/11).

**Table 2:** Change in outcomes

	Pre		Post				Follow-up			
	Mean	SD	Mean	SD	Cohen’s *d*	95% CI	Mean	SD	Cohen’s *d*	95% CI
BDI-II	23.79	10.21	13.62	7.35	1.12	0.27, 1.98	15.18	7.74	0.92	0.04, 1.8
SCS-J	60.23	15.48	67.3	15.02	− 0.46	− 1.27, 0.34	65.54	15.76	− 0.34	− 1.18, 0.5

#### Evaluation of feasibility and acceptability

The rate of intervention completion was 93%. All completers answered ‘yes’ to both the questions about satisfaction and the intent to continue the use. Regarding improving method, three of the completers requested a longer program because they wanted to learn more about the CBT and compassion training. The individual who dropped out reported that he quit the program due to a health issue and inability to attend the session. All sessions adhered to the protocol and session checklist.

### Discussion

This study was the first trial to evaluate the feasibility of CBT with compassion training in Japan. Regarding feasibility, although a participant dropped out before starting the first session, the rate of intervention completion, patient’s satisfaction, and the intent to continue the use were high. Adherence to the protocol was also satisfactory. Although some participants requested a longer program, it seems to be related to their satisfaction and motivation. Therefore, we can infer that our program has satisfactory feasibility and acceptability.

We reported that the previous GCBT had a similar situation, and the effect size was 0.80 in BDI-II [27]. Although it seems that there are differences in severity and the number of participants in this study compared to the previous study, we can infer that the new program might have a higher effectiveness. The previous study reported that the effect-size of the GCBT for depression conducted in Japan was (Cohen’s *d*) 0.53 on the BDI [28]. In that report, the program included 12 sessions of 1.5 h each. Moreover, the mean duration of major depression was 16.45 months. We may be able to conclude that our program had a larger effect size than the previously reported GCBT, although our program is shorter and the participants had severe chronic depression. This comparison might indicate that our goal was partly achieved. Furthermore, the effect size at 6-month follow-up was also high.

On the other hand, the scores on the SCS-J did not decrease significantly. This might be related to a lack of time to develop compassion. In the previous studies, compassion works took longer time than our program did [8, 16]. More time may be required to increase the level of self-compassion. However, our program has shown higher effect size than previous reports in symptoms of depression. In comparison to the general GCBT we reported before, we replaced a part of time used for cognitive re-construction and problem-solving with psycho-education of perfectionism and shame, recalling memories of compassion, and compassion letter to the self. Psycho-education of perfectionism and shame based on CFT theories might enable patients to understand their dysfunctional thoughts in more detail and might improve depressive symptoms. If more time was taken to develop compassion, the scores on the SCS-J might have increased, and depressive symptoms might have further eased. Therefore, these hypotheses should be validated in the future studies using qualitative studies focusing on the therapeutic interactions of the program.

### Limitations

First, the effectiveness of our program must be verified by conducting controlled studies. Second, the sample size of this study was small and sampling biases including sex differences might be huge. Third, we did not control for the medication. Fourth, the quantitative advantages or disadvantages of compassion works have not been clarified in this study. Fifth, the training and assessment of therapist adherence and evaluation of acceptability should be refined by using more stringent procedures.

## Additional files

**Additional file 1.** Case-formulation of CFT used in this study: the model from Gilbert and Procter [16] was used in case-formulation.

**Additional file 2.** Adherence Check List: this file was used to check the adherence of intervention.

## Abbreviations

CBT: cognitive behavioural therapy
GCBT: group cognitive behavioural therapy
CFT: compassion focused therapy
CMT: compassion mind training
TREND: Transparent Reporting of Evaluations with Nonrandomized Designs
DSM-IV: the Diagnostic and Statistical Manual of Mental Disorders IV
M.I.N.I.: the Mini-International Neuropsychiatric Interview
BDI-II: Beck Depression Inventory II
SCS-J: Self-Compassion scale in Japanese
