# Supplementary material for: Group cognitive behavioural therapy with compassion training for depression in a Japanese community: a single-group feasibility study
Source: BMC Res Notes. 2017 Dec 4;10:670. doi: 10.1186/s13104-017-3003-0 (PMC5716016; doi:10.1186/s13104-017-3003-0)
Supplement: Supplementary file 1 — Additional file 1. Case-formulation of CFT used in this study: the model from Gilbert and Procter [16] was used in case-formulation. [file 13104_2017_3003_MOESM1_ESM.pptx]

## Slide 1
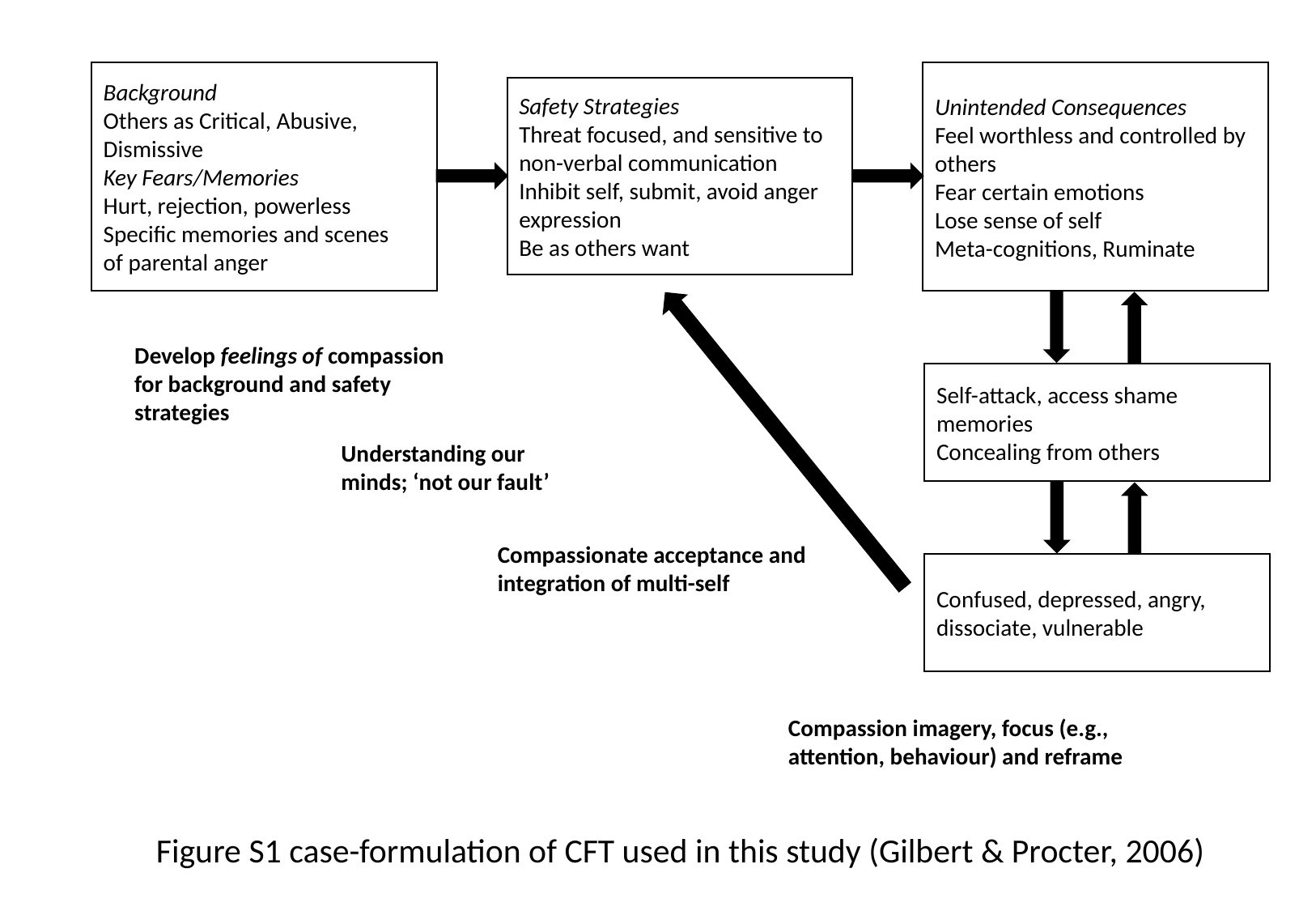

Unintended Consequences
Feel worthless and controlled by
others
Fear certain emotions
Lose sense of self
Meta-cognitions, Ruminate
Background
Others as Critical, Abusive,
Dismissive
Key Fears/Memories
Hurt, rejection, powerless
Specific memories and scenes
of parental anger
Safety Strategies
Threat focused, and sensitive to
non-verbal communication
Inhibit self, submit, avoid anger
expression
Be as others want
Develop feelings of compassion
for background and safety
strategies
Self-attack, access shame memories
Concealing from others
Understanding our
minds; ‘not our fault’
Compassionate acceptance and
integration of multi-self
Confused, depressed, angry,
dissociate, vulnerable
Compassion imagery, focus (e.g.,
attention, behaviour) and reframe
Figure S1 case-formulation of CFT used in this study (Gilbert & Procter, 2006)
