# Supplementary material for: Group cognitive behavioural therapy with compassion training for depression in a Japanese community: a single-group feasibility study
Source: BMC Res Notes. 2017 Dec 4;10:670. doi: 10.1186/s13104-017-3003-0 (PMC5716016; doi:10.1186/s13104-017-3003-0)
Supplement: Supplementary file 2 — Additional file 2. Adherence Check List: this file was used to check the adherence of intervention. [file 13104_2017_3003_MOESM2_ESM.docx]

**Adherence Check List**

**Session 1　　Introduction to GCBT and psycho-education of emotion**

□Introducing ground rules; 10min

□Psycho-education about emotions; 10min

□Introducing CBT model; 10min

□Personal examples of CBT model; 20min

□Q & A; 5min

□Presenting Homework (self-monitoring); 5min

**Session 2　　Instructions on self-monitoring as per the CBT model when distressed**

□Reflection on Homework; 10min

□Introducing the effectiveness of self-monitoring by using CBT model; 10min

□Personal examples of CBT model; 30min

□Q & A; 5min

□Presenting Homework (self-monitoring); 5min

**Session 3　　Behavioural activation**

□Reflection on Homework; 20min

□Introducing the behavioural activation and CBT model; 10min

□Brain storming of behavioural activation; 20min

□Q & A; 5min

□Presenting Homework (behavioural activation); 5min

**Session 4　　Behavioural activation and monitoring of cognitions when distressed**

□Reflection on Homework; 15min

□Introducing the cognition when distressed; 10

□Personal examples of CBT model; 25min

□Q & A; 5min

□Presenting Homework (Behavioural activation and self-monitoring of cognition); 5min

**Session 5　　 Challenging one’s own negative cognitions**

□Reflection on Homework ; 25min

□Challenging cognition; 25min

□Q & A; 5min

□Presenting Homework (self-monitoring of cognition); 5min

**Session 6　　Challenging one’s own negative cognitions**

□Reflection on Homework ; 25min

□Challenging cognition and reflection on own tendency; 25min

□Q & A; 5min

□Presenting Homework (self-monitoring of cognition); 5min

**Session 7　　 Psycho-education for perfectionism**

□Reflection on Homework; 25min

□Psychological function of perfectionism; 10min

□Personal examples of perfectionism; 15min

□Q & A; 5min

□Presenting Homework (self-monitoring of perfectionism); 5min

**Session 8　　Working with shame and self-criticism**

□Reflection on Homework; 15min

□Psychological function of shame and self-criticism; 15min

□Personal examples of shame and self-criticism; 20min

□Q & A; 5min

□Presenting Homework (self-monitoring of shame and self-criticism); 5min

**Session 9　　Recalling memories of compassion**

□Reflection on Homework; 15min

□Psychological function of compassion; 10min

□Personal memories of compassion; 20min

□Q & A; 5min

□Presenting Homework (recalling memories of compassion); 5min

**Session 10　　Compassion letters to self and other participants and relapse prevention**

□Reflection on Homework; 5min

□How to write compassion letters; 5min

□Writing compassion letters; 20min

□Relapse prevention; 20min

□Q & A; 5min

□Closing ceremony; 5min
